# The Role of Inflammation in Depression and Fatigue

**DOI:** 10.3389/fimmu.2019.01696

**Published:** 2019-07-19

**Authors:** Chieh-Hsin Lee, Fabrizio Giuliani

**Affiliations:** ^1^Neuroscience and Mental Health Institute, University of Alberta, Edmonton, AB, Canada; ^2^Division of Neurology, Department of Medicine, University of Alberta, Edmonton, AB, Canada

**Keywords:** psychoneuroimmunology, depression, fatigue, autoimmune diseases, inflammation

## Abstract

Depression and fatigue are conditions responsible for heavy global societal burden, especially in patients already suffering from chronic diseases. These symptoms have been identified by those affected as some of the most disabling symptoms which affect the quality of life and productivity of the individual. While many factors play a role in the development of depression and fatigue, both have been associated with increased inflammatory activation of the immune system affecting both the periphery and the central nervous system (CNS). This is further supported by the well-described association between diseases that involve immune activation and these symptoms in autoimmune disorders, such as multiple sclerosis and immune system activation in response to infections, like sepsis. Treatments for depression also support this immunopsychiatric link. Antidepressants have been shown to decrease inflammation, while higher levels of baseline inflammation predict lower treatment efficacy for most treatments. Those patients with higher initial immune activation may on the other hand be more responsive to treatments targeting immune pathways, which have been found to be effective in treating depression and fatigue in some cases. These results show strong support for the hypothesis that depression and fatigue are associated with an increased activation of the immune system which may serve as a valid target for treatment. Further studies should focus on the pathways involved in these symptoms and the development of treatments that target those pathways will help us to better understand these conditions and devise more targeted treatments.

## Introduction

Depression affects more than 168 million people worldwide and is one of the major causes of disease burden, accounting for the fifth highest global years lived with disability; this rate rises to the third highest in high income countries given the higher rate of prevalence (1, 2). Depression is also one of the key factors for impaired quality of life in patients affected by chronic diseases (3). In diseases such as multiple sclerosis (MS), it has also been linked to increased suicidality, which accounts for up to 7.5 times higher portion of death in MS patients than in the age-matched general population (4–6). Fatigue, defined as “a subjective lack of either physical and/or mental energy that… [interferes] with usual and desired activities” (7), is strongly associated with mental health symptoms such as depression and anxiety (8, 9). Fatigue often arises in chronic conditions and can have a prevalence as high as 99% as seen in cancer patients (10). Fatigue is one of the most debilitating symptoms of MS, with 69% of patients rating it as one of their worst symptoms and 60% reporting that it makes their other symptoms worse (11). Fatigue is also strongly linked to a worsening of one's quality of life (12, 13).

The most recent literature has shown an undeniable relationship between the activity of the immune system and neurological changes, along with subsequent psychological symptoms (14). One of the main focuses of this field is the role of the immune system in mental health and psychological disorders. Immune-mediated diseases of the central nervous system (CNS), such as MS (15), and disease modifying therapies that affect the immune system such as interferons (16) are good models to explore this association. Studies have extensively probed these interactions and found that subjects with depression and fatigue have higher levels of inflammatory immune activation, along with a host of other immunological changes (17, 18). These changes can, among other things, be used to predict treatment efficacy and future fluctuations in patient well-being.

While over the years there has been a significant amount of scientific literature on depression and fatigue (17, 19–23), there is emerging new evidence on the role of depression and fatigue in immune-mediated disorders. Here, we will review the existing knowledge regarding the links between immune response, psychological well-being, and structural changes in the brain. We will then analyze the literature regarding the presence of depression and fatigue in immune-mediated disorders. We will look at the relationship that depression and fatigue have with their existing treatments including those that do not specifically target the immune system. We will conclude by discussing some of the difficulties encountered in this line of experimentation and provide direction for potential future research.

## Immune Response and Depression and Fatigue

Early observations about the link between the immune system and psychological responses occurred in the context of cytokine-induced sickness behavior and immunotherapies such as interferon alpha (IFNα) in the context of hepatitis C treatment (24, 25). Cytokine-induced sickness behavior is a syndrome characterized by decreased activity, depression, and loss of energy because of the increased circulating levels of proinflammatory cytokines. It has been explored as a model for the role that the immune system plays in behavioral changes in both animals and humans (19, 26). The inflammatory immune response and cytokine levels have been associated with both depression and fatigue in a large body of literature across different disorders (10, 27–32). Another early line of research involved IFNα therapies, which activate an inflammatory antiviral response and are used clinically as a treatment for hepatitis C (33). Renault et al. (24) found that 17% of patients treated with IFNα developed psychiatric side effects, but also noted that the symptoms improved with the cessation of treatment. However, a recent study found that patients who suffered from depression after IFNα treatment had a significantly higher risk of having recurrent depressive episodes, which suggests that these mood changes are not a transient phenomenon but more similar to normal recurrent depressive episodes (34). The same effect on mood has also been shown with similar treatments in other disorders, such as melanoma, and Capuron et al. (33) found that these changes responded to antidepressant treatment.

**Figure 1:**
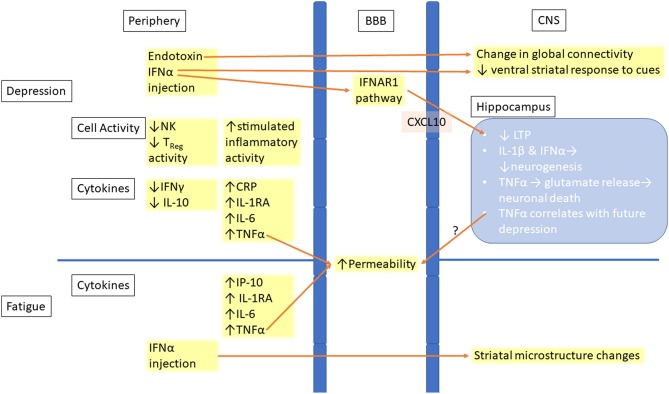
Links between peripheral inflammation and changes in the CNS in depression and fatigue. Increased inflammation is seen in the periphery in both depression and fatigue. This inflammation leads to increased permeability of the BBB, allowing for easier entry of inflammatory molecules or immune cells into the CNS. Inflammatory signaling in the CNS leads to both structural and functional changes, with the hippocampus being the location of many of the changes. BBB, Blood–brain barrier; CNS, Central nervous system; CRP, C-reactive protein; IFN, Interferon; IFNAR1, Interferon-alpha/beta receptor alpha chain; IL, Interleukin; IP-10, Interferon gamma-induced protein 10; TNF, Tumor necrosis factor; NK, Natural killer cell; T_reg_, Regulatory T cell; LTP, Long-term potentiation.

Previous meta-analyses have shown an increase in proinflammatory cytokines, such as TNFα and IL-6 (27), in people suffering from depression (Figure 1). In a more recent, larger scale meta-analysis a greater range of changes have been described in people with depression, including higher levels of TNFα, IL-6, IL-13, IL-18, IL-12, IL-1RA, and sTNFR2, along with a decrease in the proinflammatory cytokine IFNγ (18). A wide variety of chemokine levels have also been demonstrated to be significantly affected, including increased CCL2 (MCP-1), CXCL4, and CXCL7, with CCL4 having significantly lower levels in serum (31, 35). Studies found increased levels of serum IL-1RA, IL-6, TNFα, and IP-10 in cancer patients with fatigue (29, 36). There is also evidence that these changes may be predictive of future depression. A longitudinal study showed that people with higher IL-6 at age nine are more likely to have depression at age 18 in a dose dependent manner, even adjusting for a variety of factors (37). Gimeno et al. (38) conducted a study in adults that showed similar results, with CRP and IL-6 levels at baseline predicting cognitive symptoms of depression 12 years later.

Other findings indicate higher levels of TNFα and IFNγ in *in vitro*-stimulated CD8+ T cells isolated from patients with depression and IFNγ levels correlate with the severity of the condition (39, 40). In contrast, a suppression of immune responses has also been described in patients with depression (41). An early meta-analysis found that patients with depression have a higher leukocyte number and CD4+/CD8+ ratio, as well as lower natural killer (NK) cell count with impaired T and NK cell activity (17). There are a limited number of studies exploring the seemingly conflicting findings of immune activation and suppression in depression. More recent studies have shown that both can occur in the same patient, with NK and regulatory T cell (T_Reg_) activity suppressed and inflammatory monocytes activated (42, 43).

The depressive symptoms resulting from IFNα treatment, and especially the evidence suggesting that it has a long-term effect, is strong evidence for a causal link between inflammatory activation and depression. In addition, further evidence is provided by other studies showing that higher IL-6 levels predict future development of clinical depression. One of the potential mechanisms for these changes in the periphery is an increased activation of inflammatory monocytes and T cells and a higher CD4+/CD8+ ratio, which is coupled with supressed T_Reg_ activity. This combination of higher inflammatory activation and less anti-inflammatory inhibition results in a more proinflammatory peripheral environment seen in patients with depression and fatigue.

## Inflammation and Changes in the Brain

The role of inflammation in depression and fatigue has led researchers to examine the effects that peripheral inflammation has on the CNS. Some changes occur at the level of the blood brain barrier (BBB), which separates the CNS parenchyma from the peripheral blood circulation. TNFα cause changes in the endothelial cells constituting the BBB, resulting in reduced tight junction protein expression, larger extracellular gaps and increased permeability in animal models and *in vitro*, all of which are restored by treatment with anti-inflammatory drugs (44, 45) (Figure 1). An increase in proinflammatory cytokine levels including TNFα have occurred in patients who have suffered from a myocardial infarction and is associated with disruption of the BBB integrity in animal models and elevated rates of depression (46). CNS inflammation has also demonstrated that it disrupts the BBB in both MS and its animal model, experimental autoimmune encephalitis (EAE), allowing for easier entry of both cytokines and immune cells into the brain (47, 48). This increased permeability of the BBB may be one of the reasons why patients with immune-mediated diseases like MS have worse psychological symptoms compared to those with other chronic disorders.

Inflammatory changes in the brain parenchyma have also been associated with depression. Increased levels of TNFα in the hippocampus and striatum have been associated with anxious and depressed behavior in EAE studies, with the changes in the striatum occurring before the onset of clinical symptoms (49, 50). IL-1β has shown to decrease neurogenesis *in vitro* in human hippocampal progenitor cells, a common finding in depression, via activation of the kynurenine pathway; this effect being partially rescued by both inhibitors of this pathway and traditional antidepressants (51, 52).

At a cellular level changes with TNFα inducing release of glutamate by activated microglia *in vitro*, leading to excitotoxic damage in the surrounding neurons have also been reported in the literature (53). Type I interferons act through the interferon receptor chain 1 pathway in mouse BBB epithelial cells to cause impairment of long-term potentiation in hippocampal neurons *in vivo*, leading to depressive-like behaviors (54). These changes suggest a potential mechanism for the immune system's role in inducing neurological and psychological symptoms even in the absence of an altered BBB integrity.

Studies also examined the effect on the brain structure of immunotherapies associated with depression (Figure 1). IFNα treatment in patients with hepatitis C changed striatal microstructure, measured by MRI techniques such as quantitative magnetization transfer (qMT), as early as 4 h after injection, and these changes predicted development of fatigue 4 weeks later (30). Another study found that changes in brain global connectivity, which were correlated with mood changes, also occurred within 4 h from the injection of IFNα (55). Infusion of endotoxins, which also induce an inflammatory response, resulted in increased depressive mood and reduced ventral striatal response to reward cues. This indicates anhedonia, a key symptom of depression (56).

Overall, inflammation causes disruptions in the BBB along with cellular and structural changes within the CNS. *In vitro* and *in vivo* animal models have shown that inflammation decreases neurogenesis in the hippocampus, induces glutamate release from microglia, and impairs LTP. Human MRI studies have shown that IFNα and endotoxin treatments result in rapid changes in white matter structure, brain global connectivity, and functional activation, all of which are linked to depression and fatigue.

## Immune Activation Is Associated With Depression and Fatigue

Higher rates of depression and fatigue have been shown across a broad range of conditions associated with activation of the immune system such as allergies, autoimmune diseases (Type 1 diabetes, multiple sclerosis, systemic lupus erythematosus, and rheumatoid arthritis), and infections (sepsis). Patients with both atopy and asthma have a roughly 50% increased rate of depression (57, 58). Du et al. (59) found that 35.9% of asthmatic patients suffer from depression and that TNFα levels were significantly higher in the depressed cohort, with IFNγ being significantly lower.

In diabetes, activated inflammatory immune response is implicated in its pathogenesis, with immune activation being involved in the development of both type 1 and type 2 diabetes (60). Meta-analyses have found that the prevalence of depression in patients with diabetes is up to twice that of people without the disease (61, 62). Associations have been shown between depression and serum levels of CRP, IL-1β, IL-1RA, and MCP-1 in type 2 diabetes patients, with all serum levels being significantly higher in those who are depressed (63).

A meta-analysis showed that 30% of patients with systemic lupus erythematosus (SLE) suffer from depression using the standard Hospital Anxiety and Depression Scale subscale for depression (HADS-D) (64). Studies have also demonstrated that higher levels of fatigue are associated with increased risk of depression and that there is no association with disease severity in patients with SLE (65, 66). A review by Schmeding and Schneider (67) found that up to 92% of patients with SLE are fatigued, without correlation with disease severity. Significantly higher TNFα and lower IL-10 levels have been shown in depressed SLE patients and have been associated with worse depression scores (68, 69).

Depression also has a high prevalence in patients with rheumatoid arthritis (RA). Studies showed a 74% increased risk of depression compared to controls with a prevalence as high as 73.2%, and a meta-analysis found that 16.8% of RA patients suffer from it (70–72). Up to 80% of patients who are diagnosed with RA experience clinically relevant fatigue (73). Kojima et al. (74) showed that there was a positive correlation between CRP levels and depression severity in RA patients. Serum CRP levels along with erythrocyte sedimentation rate (ESR), a marker for the severity of inflammation, also have a significant correlation with fatigue (75). A Cochrane review examined a variety of anti-TNF and other biologic agents used in RA and found that they had significant effects on the fatigue experienced by patients, further strengthening the suggestion that fatigue may in part related to immune responses (76).

Patients with MS have a lifetime prevalence of 25–50% for depression, with an incidence rate ratio of 2.41 compared to age- and sex-matched controls (77–79). An increase in the incidence and prevalence of depression, along with an increase in the rate of prescriptions for antidepressant, occur as early as 2 years before MS diagnosis (80, 81). The prevalence of fatigue is even higher than that of depression, with a prevalence as high as 75% (82–85). In later phases of MS the prevalence of fatigue can increase up to 95% (86). However, there is a large variability in results regarding the role of immune activation in depression and fatigue in MS patients, with studies describing contradictory results. Some studies have demonstrated an increase in peripheral blood cell-derived TNFα mRNA along with circulating TNFα and IFNγ in MS patients with fatigue (87, 88). Brenner et al. (89) also showed that higher CSF IL-6 levels are significantly associated with both increased depression and fatigue scores. Alternatively, a study by Malekzadeh et al. (90) found that TNFα and IFNγ, along with 10 other cytokines, did not vary significantly between fatigued and non-fatigued patients, although the study did find significant correlation with IL-6 levels. In contrast, Giovannoni et al. (91) showed that circulatory CRP and sICAM-1 levels are not correlated with fatigue.

The link between immune activity and depression and fatigue is not only shown in immune related disorders but also in cases where the immune system is activated in response to infections. Sepsis is a systemic immune response to an infective agent which leads to broad proinflammatory activation. Even after the resolution of the condition, survivors have a persistently higher concentration of circulating inflammatory markers and a range of long-term symptoms leading to decreased quality of life (92–94). Davydow et al. (95) found that while survivors of sepsis have a higher prevalence of depression compared to the general population, this was not significantly higher than that preceding the infection. This high prevalence of depression in patients pre-sepsis is consistent with other findings that demonstrate psychosocial stress increases depression and immune activation (96) and is associated with a greater short-term risk of sepsis (97). There have been very few studies on post-sepsis depression in humans, however, studies in animal models have shown sepsis-like conditions leading to affective changes (98). These studies in animal models have also found that immune suppression, by way of dexamethasone or by inhibiting the NF-κB pathway, reduces the resulting depressive-like behavior in the animals (98, 99). There may be a potential role for the “priming” of the immune system by condition such as sepsis or treatments like IFNα, which show an increased risk of developing depression later on (34). Further studies are needed to establish whether previous immune activation primes the immune system to be more sensitive to stress or other insults, leading to an increased risk of depression and fatigue in the future.

## Immunomodulatory Effects of Antidepressant and Anti-Fatigue Therapies

Changes in the levels of immune markers have also been associated with the response to antidepressant therapies and found helpful in predicting treatment efficacy (Table 1). In mice treated with LPS, serotonin reuptake inhibitor (SSRI) and serotonin–norepinephrine reuptake inhibitor (SNRI) administration lead to decreased serum levels of TNFα and increased levels of IL-10 (104). In the repeated social stress model, treatment with tricyclic antidepressant (TCA) decreased microglial expression of IL-6 mRNA both *in vivo* and following *ex vivo* stimulation, where TNFα and IL-1β mRNA levels were also reduced (111). *In vitro* studies using animal macrophages have also confirmed similar immunosuppressive effects where the decrease in IL-6 and increase in IL-10 that follows treatment with amitriptyline, fluoxetine, and mianserin, suggests that such effects may be mediated by an inhibition of the nuclear factor kappa-light-chain-enhancer of activated B cells (NF-κB) pathway (105). On the other hand, Munzer et al. (109) found that treatment *in vitro* of whole blood cultures with SSRIs and mirtazapine, a tetracyclic antidepressant (TeCA), had the opposite effect on the stimulated production of cytokines, with an increase in inflammatory markers including IL-1β, IL-6, and TNFα.

**Table 1:** Efficacy prediction and immunomodulatory effect of therapies.

	**Efficacy prediction from immune markers**	**Immunomodulatory effect**
SSRI	Human: no predictive effect (100) Human: lower CRP, IL-6, and TNFα predict better efficacy (101–103)	Animal: decrease in serum TNFα and increase in IL-10 (104) Animal: anti-inflammatory effect in macrophage via NF-κB pathway (105) Human: decrease in serum IL-1β, IL-2, IL-4, IL-5, IL-6, IL-10, IL-17A, and TNFα; increase in IFNγ (100, 101, 106–108) Human: increased inflammatory activation (109)
SNRI	Human: lower IL-6 predicts better efficacy (101)	Animal: decrease in serum TNFα and increase in IL-10 (104) Human: decrease in serum IL-1β, IL-2, IL-4, IL-5, IL-6, IL-8, IL-10, IFNγ, GM-CSF, and TNFα (101, 108)
TCA	Human: higher CRP and lower IL-6 predict better efficacy (103, 110)	Animal: decrease in inflammatory activity in splenocytes and microglia; decrease in serum IL-1β (111) Animal: anti-inflammatory effect in macrophage via NF-κB pathway (105) Human: significant decrease in TNFα in responders (110)
Ketamine	Human: lower FGF-2 and IL-1RA predict better efficacy (112)	Human: transient decrease in G-CSF, IL-13, and IP-10; 24-h increase in IL-7 and decrease in IL-8 and PDGF-AA (112)
Sleep deprivation	Human: lower IL-6 predicts better efficacy (113)	
ECT	Human: lower TNFα at first ECT predict better efficacy (114)	Human: acute increase of IL-1 and IL-6; long-term decrease of TNFα and IL-6 (115, 116) Human: transient increase in natural killer cell activity (117)
Psychotherapy		Human: decrease in IFNγ from stimulated PBMC (39)
Exercise	Human: higher TNFα predicts better efficacy (118)	Human: correlation between decrease in IL-1β and depression (118)
Amantadine		Animal: no effect on splenocyte expression of IFNγ or IL-10 (119)
	**Efficacy prediction from immune markers**	**Immunomodulatory/treatment effect**
**IMMUNE TARGETING THERAPIES**		
Minocycline		Animal: antidepressant effect with increase in IL-10, IL-15, and VEGF in the brain (120, 121)
Anti-TNF	Human: higher CRP, TNFα, and sTNFR linked to better efficacy (122)	Human: anti-fatigue effect in RA and sarcoidosis (76, 123) Human: antidepressant effect with significantly greater CRP decrease in responders (122, 124)
Anti-IL-6		Human: antidepressant effect (124)
Dexamethasone		Animal: immune suppression effective in sepsis model (99) Human: anti-fatigue effect with prophylactic treatment (125)
B cell depletion		Human: anti-fatigue effect in RA (126)

*Summary of the interaction with the immune system of various antidepressant and anti-fatigue treatments, with the predictive efficacy of immune markers and their immunomodulatory effect listed. The experimental model used in each study (i.e., human vs. animal) is noted. SSRI, Selective serotonin reuptake inhibitor; SNRI, Serotonin–norepinephrine reuptake inhibitor; TCA, Tricyclic antidepressant; ECT, Electroconvulsive therapy; TNF, Tumor necrosis factor; sTNFR, Soluble tumor necrosis factor receptor; CRP, C-reactive protein; FGF, Fibroblast growth factor; IL, Interleukin; IP-10, Interferon gamma-induced protein 10; NF-κB, Nuclear factor kappa-light-chain-enhancer of activated B cells; IFNγ, Interferon-γ; G-CSF, Granulocyte colony-stimulating factor; GM-CSF, Granulocyte-macrophage colony-stimulating factor; PBMC, Peripheral blood mononuclear cell; PDGF, Platelet-derived growth factor; VEGF, Vascular endothelial growth factor; RA, Rheumatoid arthritis*.

Meta-analysis of human studies examining changes in a variety of serum cytokine levels showed that treatment with antidepressants lowered levels of IL-1β (the studies disagree on whether this is present only in SSRIs or also other antidepressants), IL-4, IL-6, and IL-10 (106, 107). Other studies have also demonstrated that antidepressants have different immunomodulatory activities. Chen et al. (108) found that an SNRI (venlafaxine) had greater anti-inflammatory activity when compared to an SSRI (paroxetine). This study also showed that treatment with SSRIs significantly increase IL-6 levels and led to a non-significant increase in TNFα levels, contrary to previous findings. Human studies have also shown that treatment with psychotherapy has similar immunomodulatory effects to that of pharmaceutical therapies (39).Other recent studies have also looked at exercise, transcranial direct current stimulation (tDCS), and standard of care treatment and shown that the levels of a variety of circulating cytokines generally decrease following treatment, although there is no agreement on the correlation with improvement of depressive symptoms (100, 118, 127). Treatments such as electroconvulsive therapy (ECT) have somewhat similar effects on the immune system, although with different characteristics. Overall, ECT is associated with an initial spike of IL-1 and IL-6, with the levels of TNFα and IL-6 falling after treatment over the long term, though these results come from a limited number of studies (115). One study looked at the effect of ECT as an adjunctive treatment to antidepressants and found that while it did cause a significant decrease in IL-6, TNFα levels increased with treatment (116). ECT has also been shown to reverse the change in NK cell activity, which is decreased in depressed patients (17, 117).

Studies have also illustrated that immune markers may be used to predict treatment efficacy. Lower baseline levels of proinflammatory cytokine predict better treatment response to TCAs, SSRIs, TeCAs, and ketamine, with responders having a significant decrease in these cytokine levels (110, 112, 128). However, Uher et al. (103) showed that baseline CRP levels predicted a differential treatment response to different antidepressants. Those patients with lower levels of CRP respond better to the SSRI escitalopram, while those with higher levels had a better response to nortriptyline, a TCA. These observations suggest that the clinical effects of SSRIs may be at least partially due to anti-inflammatory effects, which may not be the case for tricyclics. Higher IL-6, but not TNFα, levels in patients have also been associated with worse treatment efficacy of multiple different SSRI and SNRI treatments (101). On the other hand, Eller et al. (102) found that higher TNFα levels were associated with treatment non-response in patients being treated with escitalopram.

In antidepressant sleep deprivation therapy, higher IL-6 levels predicted worse treatment response in depressed patients with bipolar disorder, in agreement with previous studies on antidepressants (113). Lower TNFα levels at the first ECT have also shown to predict better treatment outcome (114). However, this correlation between higher inflammatory cytokine levels and worse treatment efficacy is not found in all treatments. It has been shown that higher serum proinflammatory cytokine levels, in this case TNFα, predicts a positive response to exercise therapy (118). The differences in predictive effects of circulating inflammatory cytokine levels regarding the efficacy of different treatments suggest that their mechanisms may differ, with anti-inflammatory effects being more important for some treatments, such as SSRIs, than others.

Few drugs are effective in treating fatigue; with even less studies done on the interaction those drugs have with the immune system. Amantadine is one drug that has been effective in patients with MS (129) but there is however a lack of studies on its immunomodulatory effect. A study on the effect of amantadine treatment in rats showed that while it enhanced the effect of fluoxetine when co-administered, it did not change the expression of IFNγ or IL-10 levels by splenocytes (119). Further studies will be required to examine whether its efficacy as a treatment for fatigue in MS patients is through effects on the immune system or through other pathways.

## Effects on Depression and Fatigue by Treatment Targeting the Immune System

As the immune system plays a role in depression and fatigue, anti-inflammatory drugs and other treatments that change the immune system serve as a potential treatment option (Table 1). An earlier meta-analysis of anti-inflammatory medications showed that there is a potential effect of COX-2 inhibitors on depression, with cytokine inhibitors having no significant effect. However, the authors were cautious in their conclusions due to the high heterogeneity of the studies (130). The use of non-steroidal anti-inflammatory drugs (NSAIDs) as an add-on to standard antidepressant therapy should however be done carefully due to the role innate immune response plays in normal neurological functions (23), and especially since the antidepressant effect of SSRIs can be attenuated by anti-inflammatory treatment (131). Minocycline, an antibiotic with immunomodulatory effects, has also been found to have antidepressant effects (120, 132). One potential pathway for its action is through the rescuing effect on mouse hippocampal neural stem cell proliferation, which is suppressed by IFNα (133). A small meta-analysis of three Randomized Control Trials (RCTs) also suggest that it has a large treatment effect for depression and should be studied further (121). Given that minocycline may be effective in treating MS and lowering the risk of conversion from clinically isolated syndrome to MS (134, 135), it could serve as an effective adjunctive treatment for patients with MS who are suffering from depression, though more studies will be required to support this hypothesis.

More recent studies suggest that the antidepressant effect of drugs targeting cytokines is significant (136). A meta-analysis by Kappelmann et al. (124) found that anti-cytokine drugs are significantly more effective than placebo in the treatment of depression. An RCT conducted by Raison et al. (122) examined the efficacy of TNFα antagonists in treatment-resistant depression and showed that, while no change was seen in the overall group, there was a significant effect in those with higher baseline CRP levels. The responders in this trial also had higher baseline plasma TNF and soluble TNF receptor levels and exhibited a significantly greater decrease in CRP than non-responders. This suggests that while the targeting of the immune system for treatment of depression may not work in all patients, it is a valid target for a subset of depressed patients whom inflammation may play a major role. The targeting of IL-6 by the IL-6 receptor antagonist tocilizumab has also been shown to improve depressive symptoms (124). Given that a third of depressed patients are treatment resistant even after four successive treatment steps (137), the exploration of the immune system as a treatment target is a legitimate area of interest, especially in those with higher baseline inflammation. Studies have also targeted the immune system through other means, including miR-155, a microRNA that is involved in inflammation and neuroplasticity (138). A study by Fonken et al. (139), found that mice with miR-155 KO in the hippocampus presented less depressive-like behavior and had significantly lower IL-6 and TNFα expression in this area. The increase in NFKBIA, a NF-κB inhibitor, expression in females in this study along with findings from *in vitro* studies (105) suggest that the NF-κB pathway's role in inflammatory activity may play a part in the development of depression, making it a potential treatment target to be explored. For treatment of fatigue, Elfferich et al. (123) showed that treatment with anti-TNFα drugs improved fatigue in sarcoidosis and had significantly better efficacy compared to both control and treatment with prednisone, a more general anti-inflammatory drug. A study in colorectal cancer patients found that prophylactic use of dexamethasone, which has anti-inflammatory effects, led to significantly lower levels of fatigue and better treatment tolerance compared to untreated control patients (125). Patients with RA who were treated with rituximab, an antibody which targets and depletes B cells, have also reported an improvement in fatigue after 1 year of treatment (126). On the other hand, a study examining chronic fatigue syndrome (CFS) showed that treating fatigue may not always be so straight forward (140). The authors targeted IL-1, which has been linked to CFS, using a receptor antagonist and found no significant effect on fatigue. The study did not measure cytokine levels in patients, so it is unclear whether patients with higher baseline IL-1 would have benefited more from the treatment, which would be inline with the results shown by Raison et al. (122).

Overall, there is strong evidence that changes in the immune system may be one of the pathways through which antidepressant therapies act. Many of the pharmaceutical antidepressant agents reduce inflammatory activation in immune cells and lower circulating inflammatory cytokine levels. Other treatments such as ECT, tDCS, psychotherapy, and exercise also result in decreases in inflammatory cytokine levels. Lower baseline inflammatory cytokine levels are also shown to predict better efficacy in most types of antidepressant treatments, except for exercise. Anti-inflammatory treatments have also been shown to be effective, with medications such as NSAIDS and anti-cytokine drugs having antidepressant effects. While the anti-fatigue drug amantadine has not been shown to have immunomodulatory effects, drugs targeting of TNFα and B cells both lead to decreased fatigue, suggesting potential targets for drug discovery for anti-fatigue therapies.

## Future Directions

While there is consensus on the presence of a relationship between the immune system and symptoms like depression and fatigue, there are still some unanswered questions. One of these questions is the role this relationship plays in specific disorders such as MS, where the findings are less clear. In the case of some chronic diseases, such as MS, both depression and fatigue are hard to diagnose. This is due to the overlapping symptoms and the difficulty in determining what is caused by the disease itself (primary) and what is a result of a reaction to the diagnosis and disability induced by disease or the effects of its treatment (secondary). The complexity of depression and fatigue, both of which have multiple causes, makes studying these symptoms challenging. The above issues are further compounded in immune-related disorders, where there is a relative dearth of studies examining the immune-psychological relationship, making it more difficult to draw a conclusion from the contradictory findings (78, 141). The contradictions in the results, the limited studies on this topic, along with the need to better understand the complex conditions that deeply affect the patient's suffering from depression and fatigue, demonstrates a vital need for further comprehensive studies.

Another difficulty when comparing studies on inflammation in depression and fatigue is the lack of comprehensive analysis of different cytokines in most studies. Many of the studies only look at a small subset of cytokines, and these subsets are often different between studies. This is less problematic in conditions where there is agreement on the affected markers, like cancer, but it can be an issue in diseases where there is no clear consensus, such as MS. In conditions with no clear consensus, studies should aim to measure a wider range of markers to make sure the potential changes are discovered, which would also help with reviewing results in the future and allowing for better conclusions to be made.

Studies should also explore aspects of the immune system beyond the often-measured level of circulatory cytokines and include the less common *in vitro* activation assays to explore other facets of the pathways. The study by Blank et al. (54) serves as a good example by examining changes in the whole pathway, covering immune, endothelial, and neural cells along with behavioral changes and treatment effects. Studies such as these paint a more comprehensive picture of how the immune system exerts its effect on the brain, which will also help to discover potential drug targets for treatment. Future studies should also explore potential drug targets based on known changes that result from depression and fatigue. This is important for discovering new antidepressant treatments but is even more important for treatment of fatigue, given that there are few existing treatments, often with unclear efficacy.

In addition, further studies should be done to examine whether previous immune activation due to sepsis or interferon treatments, for example, can independently prime the immune system. This would be similar to the two-hit hypothesis as suggested for some other psychological disorders (142), with the primed immune system making it easier for other biopsychosocial “hits” to result in increased susceptibility or increased severity of future depression and fatigue. While previous studies have shown that stress can prime the immune system and result in larger activated immune response (143, 144), none have looked at the clean effect of intense immune activation, taking out the effect of the hypothalamic–pituitary–adrenal (HPA) axis, and the role it plays in future depression. This would be an interesting direction to explore as it would inform physicians to keep careful track of patients who have previously had strong activation of the immune system, since they may be more susceptible to suffering from depression and fatigue.

## Conclusion

Depression and fatigue are symptoms that significantly impair those who suffer from them and it is therefore important to increase our understanding of both their etiology and the mechanisms involved. The described link between depression, fatigue and increased immune activation, the psychological effect of proinflammatory insults, and the treatment efficacy of anti-inflammatory medications, provide convincing evidence supporting the hypothesis that inflammation plays a role in the causation of some forms of depression and fatigue. However, in some diseases, such as MS, there is still conflicting evidence. For the disorders where the link is unclear such as immune-mediated diseases, a greater number of comprehensive, high quality studies are required to help better understand the immune-neuro-psychological interactions. Further exploration of this relationship between the immune and psychological systems will improve our understanding of the disease conditions and assist in designing better treatments to improve the quality of life of individuals affected by depression and fatigue.

## Author Contributions

C-HL wrote the first draft of the manuscript. FG provided supervision and assisted with the writing and content of the manuscript. All authors contributed to manuscript revision, read, and approved the submitted version.

### Conflict of Interest Statement

The authors declare that the research was conducted in the absence of any commercial or financial relationships that could be construed as a potential conflict of interest.

## Abbreviations

SSRI: Selective serotonin reuptake inhibitor
SNRI: Serotonin–norepinephrine reuptake inhibitor
TCA: Tricyclic antidepressant
ECT: Electroconvulsive therapy
BBB: Blood–brain barrier
CNS: Central nervous system
CRP: C-reactive protein
IL: Interleukin
IFNγ: Interferon-γ
NF-κB: Nuclear factor kappa-light-chain-enhancer of activated B cells
TNF: Tumor necrosis factor
MS: multiple sclerosis
RA: Rheumatoid arthritis
SLE: Systemic lupus erythematosus.
